# “It felt like I had an old fashioned telephone ringing in my breasts”: An online survey of UK Autistic birthing parents' experiences of infant feeding

**DOI:** 10.1111/mcn.13581

**Published:** 2023-11-01

**Authors:** Aimee Grant, Catrin Griffiths, Kathryn Williams, Amy Brown

**Affiliations:** ^1^ Centre for Lactation, Infant Feeding and Translation Swansea University Swansea UK; ^2^ Autistic UK, CIC Llandudno UK; ^3^ School of Social Sciences Cardiff University Cardiff UK

**Keywords:** Autism, Autism Spectrum Disorder, breastfeeding, formula feeding, infant feeding

## Abstract

Around 3% of people are Autistic. Autistic people communicate differently from non‐Autistic people and experience the sensory world differently. There is limited evidence that Autistic people can face additional barriers to breastfeeding. We are an Autistic‐led research team that developed an online survey following consultation with the Autistic community. Autistic people from the UK who had been pregnant were eligible to take part in the survey, which focused on the entire maternity journey. A total of 193 people participated, of whom 152 had experiences of infant feeding (137 breastfeeding, 82 formula feeding). Participants were highly motivated to breastfeed, and almost half of those who breastfed found it positive always or most of the time. However, breastfeeding—and in particular the milk let‐down reflex—could result in pain and sensory difficulties, including ‘feeling odd’. Expressing breastmilk always or most of the time was reported by 10% of breastfeeding participants. The intensity and unpredictability of both breast and formula feeding were challenging to manage. Parents reported that it was easy to understand how to prepare infant formula, but that it could also be a negative and anxiety‐inducing experience. Support for breast and formula feeding was often considered inadequate. When parents did access breastfeeding support, this significantly improved a range of breastfeeding experiences. However, participants recommended more tailored support and continuity of carer. To meet the needs of Autistic birthing parents, those providing infant feeding support should receive training on Autism through a neurodiversity‐affirming lens, which should be delivered by Autistic people.

## Key messages

- Many Autistic people are highly motivated to breastfeed, regardless of challenges.
- Autistic people may experience sensory discomfort when breastfeeding, including from the intensity of touch between themselves and their baby. Distraction and reducing sensory burdens can help.
- Expressing always or most of the time was relatively common (10%), and a route to maintaining exclusive breastfeeding when directly breastfeeding was too uncomfortable.
- Receiving breastfeeding support was significantly associated with improvements in a range of breastfeeding experiences.
- Breastfeeding support for Autistic people should be offered on a one‐to‐one basis with continuity of carer.

## Graphical abstract

Participants were highly motivated to breastfeed. Almost half of those who breastfed found it positive always or most of the time. However, breastfeeding could result in sensory difficulties and disrupted routines. Support for breast and formula feeding was often considered inadequate.

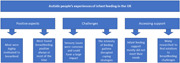

## 1 INTRODUCTION

Autism is a lifelong disability. A recent population surveillance programme in the United States identified that around one in 36 8‐year‐old are Autistic^1^ (Maenner et al., 2023). There is limited variation in Autism prevalence across gender and age, although underdiagnosis for women and those without intellectual disabilities has been common historically due to diagnostic practices (Chester, 2019; Lai & Baron‐Cohen, 2015). Being Autistic is associated with differences in communication and most Autistic people have sensory processing differences, such as experiencing the world to be too visually bright or busy and too loud (Yergeau, 2018). In line with the social model of disability (Shakespeare, 2004), these differences are *not* inherently deficits, but are frequently perceived as deficits due to environmental and social barriers that could be removed with accommodations (Woods et al., 2018), as is recommended in core legislation, such as the UK Equality Act 2010. However, Autistic people are frequently misunderstood by non‐Autistic people in what has been termed ‘the double empathy problem' (Milton, 2012). When the environment around Autistic people does not meet their needs, they become dysregulated, including feeling irritable or anxious (Beardon, 2019). Reregulation can occur through ‘stimming’, repetitive movements such as fidgeting with something in one's hands or moving their body. Without this, or if the environment or social demands are too much, Autistic people will reach the peak of being overwhelmed and either meltdown (an explosive burst of energy, such as pacing or shouting, which cannot be stopped) or shutdown (retreating into themselves, such as becoming nonspeaking or even catatonic) (Beardon, 2019). If Autistic people's needs are consistently unmet, meltdowns and shutdowns increase in frequency and can lead to Autistic burnout and self‐injurious behaviours (Cassidy, 2020).

Breastfeeding has long been established as conferring health benefits to mother and infant when compared to formula feeding (Renfrew et al., 2012). For this reason, exclusive breastfeeding for the first 6 months of life is recommended, with breastfeeding continuing alongside solid food for at least the first 2 years (World Health Organization, 2017). However, breastfeeding rates are low in many high‐income countries. For example, in the United Kingdom while four in five women breastfeed initially, the rate declines steeply and only around 1% of infants receive breastmilk exclusively for 6 months (Health and Social Care Information Centre, 2012). This is the result of numerous barriers, including mothers receiving inadequate breastfeeding support (Brown, 2021), aggressive marketing by the infant formula industry (Rollins et al., 2023) and the sexualisation of breasts leading to a hostile environment (Grant, Pell, et al., 2022).

To date, attention has been primarily focused on the relationship between maternal breastfeeding and Autism diagnosis in children (Tseng et al., 2017). However, this neglects the experiences of Autistic parents who breastfeed. This focus on Autistic children can be explained by the relative invisibility of Autistic mothers (Douglas, 2013) and 98% of Autism research funding being directed towards research on Autistic children (Nicolaidis et al., 2019). Quantitative comparative research has identified that Autistic people were more likely to have breastfed or attempted to breastfeed compared to non‐Autistic people (Hampton et al., 2023) and that there was no significant difference between groups (Pohl et al., 2020). This research has also identified inconclusive evidence regarding breastfeeding problems between Autistic and non‐Autistic birthing parents, with significant results limited to Autistic people being more likely to experience sensory issues (Hampton et al., 2023), and difficulties breastfeeding their second child (Pohl et al., 2020). That said, Autistic people found accessing breastfeeding support more difficult and were less satisfied with the support they received (Hampton et al., 2023).

We previously undertook a qualitative systematic review of Autistic mothers' experiences of infant feeding (Grant, Jones, et al., 2022), identifying only eight peer‐reviewed publications, many of which contained limited depth. The existing research highlighted that Autistic birthing parents were often very determined to breastfeed and that breastfeeding could be positive for Autistic mothers (Gardner et al., 2016). However, Autistic mothers reported many of the barriers identified in a general population of breastfeeding mothers, including a lack of support and challenges establishing breastfeeding (Pelz‐Sherman, 2014). An additional challenge for Autistic parents was navigating sensory, pain and interoceptive differences, that is internal bodily feelings such as milk let‐down, associated with breastfeeding (Burton, 2016). Furthermore, becoming a parent was reported to result in stress, exhaustion and a loss of control over routines needed to avoid Autistic burnout (Adams et al., 2021). Additionally, mothers reported a general lack of social support as a result of Autistic challenges in fitting into existing ‘mother and baby’ support groups (Hampton et al., 2021) and a lack of availability of peer support from Autistic parents (Hampton et al., 2023). In addition to these breastfeeding challenges, maternity and infant feeding services were reported to be founded on a poor understanding of Autism, meaning that services were not designed to meet Autistic parents' needs (Dugdale et al., 2021) and communicating with health professionals was more challenging than for non‐Autistic peers (Pohl et al., 2020). Furthermore, breastfeeding support models have not yet been evaluated with this group.

## 2 METHODS

Aim: to understand Autistic people's experiences of the maternity period and associated health care.

We developed an online survey aimed at Autistic people from the United Kingdom who had been pregnant, to gain a greater understanding of these issues and others impacting Autistic people in the perinatal period, including experiences of pregnancy loss, the antenatal period and birth. We also included questions on barriers to accessing health services generally and their views and opinions on ‘health passport’ tools. This paper specifically reports on data relating to infant feeding, including support accessed. Our consultation with the Autistic community when developing this study highlighted a strong preference for gender‐neutral language, which will be used throughout this article.

### 2.1 Population

The survey was targeted at Autistic people aged at least 18 years who had been pregnant and lived in the United Kingdom. Participants did not need to have received a formal Autism diagnosis, with those undergoing diagnosis at the time of the study and those self‐identifying as Autistic able to take part. The decision to include those without an Autism diagnosis was based on two factors relating to diagnostic availability. First, the known underdiagnosis of people Assigned Female at Birth (Chester, 2019). Second, the long delays in Autism diagnosis pathways in the United Kingdom are well established, with over 122,000 people currently awaiting diagnosis in England alone (NHS Digital, 2022). Furthermore, research has identified similarities between those who self‐identify as Autistic and those who are formally diagnosed (McDonald, 2020). Due to the dearth of information available on the topic, we did not put a time limit on the duration since the last pregnancy. Furthermore, because we included questions on pregnancy loss in the questionnaire, participants did not have to have experienced a live birth to take part, although those without a live birth would not have had the opportunity to respond to the infant feeding questions.

### 2.2 Survey design

An online survey was developed to understand the impact of Autistic people's impairments in relation to communication and the sensory environment and to consider how this impacted their experiences of maternity and infant feeding care. Demographic questions were asked in relation to age, gender identity, ethnicity, educational qualifications and disability. A variety of closed and open questions focused on experiences of Autism and on whether participants were diagnosed, undergoing diagnosis or self‐identifying as Autistic, communication preferences and sensory experiences, and if these changed during pregnancy. Participants provided the years in which they were pregnant and were asked a range of closed questions around care for pregnancy loss (if relevant), NHS antenatal care, supplementary antenatal services, such as NCT classes, where they gave birth and postnatal care provided. At the end of each set of closed questions, participants were directed to two open text boxes to add (i) any thoughts they would like to share about their experiences and (ii) any recommendations to improve support for Autistic people. Participants were asked to consider the impact of COVID‐19 on their care if relevant. The questions related to infant feeding were based on a qualitative systematic review and thematic synthesis of Autistic experiences of infant feeding (Grant, Jones, et al., 2022). These included questions focused on: motivation, pain, intensity, unpredictability, sensory issues, expressing breastmilk and experiences of breastfeeding support.

### 2.3 Recruitment and data collection

The survey, hosted on Qualtrics, was advertised exclusively on social media for just under 2 months (10 February 2022 to 30 March 2022). Two closed Facebook groups for Autistic parents (Autistic Breastfeeding, Chestfeeding and Bodyfeeding parents; Autistic and pregnant, parenting or planning) and one open group for Autistic adults and parents of Autistic children (Autism Inclusivity), which Grant was a member of agreed the advertisement could be shared. Autistic UK also shared the link to the survey, as well as Grant regularly advertising the survey on Facebook and Twitter, which was shared by others. The advertisement noted that there were ten £20 vouchers available, which would be awarded as ‘prizes’ for randomly selected participants.

### 2.4 Analysis

All survey responses were exported into Microsoft Excel. Respondents who did not answer the infant feeding questions (i.e.,: participants who had only experienced pregnancy loss or who did not answer all sections of the survey) were excluded from the analysis for this paper. Descriptive statistics were generated in SPSS (Version number: 28.0.1.1) by Griffiths. Kruskal–Wallis analyses were conducted to investigate whether receiving breastfeeding support and the different types of breastfeeding support received resulted in any significant differences in parents' experience of breast and formula feeding. Grant undertook inductive thematic analysis (Braun & Clarke, 2022) of the open text questions relating to breast and formula feeding facilitated by NVivo vR. This followed a six‐stage process of: becoming familiar with the data by reading and rereading; generating initial codes; searching for themes within the codes; reviewing the themes with the broader study team; defining the themes with the broader study team until a consensus was reached and writing up the findings, which were again reviewed by the broader study team. We use verbatim quotations in the reporting of the findings.

### 2.5 Reflexivity and ethics

The research team involved two Autistic researchers; the study lead Grant and Williams, the research director of Autistic UK, a UK‐based Community Interest Company led by Autistic people for Autistic people. Two other Autistic researchers reviewed the survey for its suitability for Autistic audiences. Brown is an expert in pregnancy and infant feeding; they reviewed the survey for its suitability to the UK health care context. Griffiths is a health psychologist with a background in infant feeding, who analysed the quantitative data.

The study was awarded ethical approval by the School of Health and Social Care, Swansea University. Participants were required to read a detailed information sheet on the Qualtrics platform before providing their consent to complete the survey. They were told that they could freely choose not to answer any questions beyond the eligibility screening questions. Although we collected contact details to facilitate a prize draw (if participants wished to be entered), these were removed from the data before analysis, and no other identifying information was collected. The prize draw was facilitated by assigning each email address a number (based on the order in which responses were completed) and using a random number generator. All aspects of the study were performed in accordance with the ethical standards set out in the 1964 Declaration of Helsinki.

## 3 RESULTS

Below we present our results in relation to participants' demographics, infant feeding mode, breastfeeding experiences, formula feeding experiences and experiences of accessing infant feeding support. Where quotations are provided, they are contextualised by: Diagnosis status, primary communication method, ethnicity, gender identity and highest qualification. Many participants noted that although they were primarily speaking, they would turn to nonspeaking communication methods during times of stress.

### 3.1 Demographics

Of the 193 Autistic people who completed the survey, which we considered to be completing the demographic questions and at least one other question, 152 had given birth and had experience of at least one infant feeding method. In addition to this, three participants contacted Author 1 to provide information relating to their maternity and infant feeding experiences when they did not feel they had sufficient energy to engage in a full survey. This data was added to the thematic analysis, but no demographic data was available for these participants.

Demographic details provided by these participants can be seen in Table 1. The majority of participants were: diagnosed as Autistic (57.9%), university graduates (63.1%), spoke as their primary communication mode (84.2%), identified as cis‐gendered women (82.9%) and were of white ethnicity (92.1%). Furthermore, three quarters (76.3%) noted that they were Disabled in addition to being Autistic, with 73.1% of these noting that they did not receive sufficient support for their disabilities. The mean age of participants was 36.39 years, (SD, 7.25 range 19–54). The majority (*n* = 125, 82.2%) had given birth within the last 10 years, and the mean time since their most recent birth was 6.73 years (SD: 4.96; range: 2 months–25.17 years). The mean age of all of the participants' children (i.e.,: not only from their most recent birth) was 9.51 years (SD, 6.16; range 0–28). Over two‐thirds of participants (67.7%) noted that they ‘masked’ their Autistic behaviours and communication style always or most of the time.

Participants were asked how many times they had given birth (*M* = 2.06; SD: 1.11.) 51 (33.6%) had given birth once, 63 (41.4%) twice and 26 (17.1%) three times. The remainder (12 participants, 7.9%) had more births with a range of up to seven.

**Table 1:** Demographic characteristics of participants (*n* = 152).

Demographic	Subcategories	*N*	%
Autism diagnosis status	Diagnosed	88	57.9
	Undergoing diagnosis	29	19.1
	Self‐identifying	35	23.0
Communication preference	Speaking	128	84.2
	Sign language	1	0.7
	Alternative and augmentative communication (AAC)	1	0.7
	Other	20	13.2
Autistic masking	Always or most of the time	103	67.7
	About half of the time	29	19.1
	Sometimes or never	20	13.2
Gender identity	Cis woman	126	82.9
	Nonbinary	14	9.2
	Other	10	6.6
	Prefer not to say	2	1.3
Ethnicity	White	140	92.1
	Mixed or multiple	5	3.3
	Asian or Asian British	2	1.3
	Other	4	2.6
	Prefer not to say	1	0.7
Disability in addition to being Autistic	Yes	116	76.3
	No	33	21.7
	Prefer not to say	3	2.0
Disability impact	A lot	37	24.3
	A little	84	55.3
	Not at all	26	17.1
	Prefer not to say	2	1.3
Receive sufficient support for disabilities	Strongly or somewhat agree	18	11.8
	Neither agree nor disagree	21	13.8
	Strongly or somewhat disagree	111	73.1
	Prefer not to say	2	1.3
Highest qualification	None	1	0.7
	General Certificate of Secondary Education (GCSE)	12	7.9
	A levels	17	11.2
	National vocational qualification (NVQ)	21	13.8
	Undergraduate degree	54	35.5
	Taught postgraduate degree (e.g.,: Masters, postgraduate certificate)	31	20.4
	Doctorate	11	7.2
	Other	5	3.3
Location	England	116	76.3
	Scotland	11	7.2
	Wales	20	13.2
	Northern Ireland	5	3.3
Participant's age	Mean: 36.39 years	‐	‐
	SD: 7.25		
	Range: 19–54 years		
Time since the most recent birth	Mean: 6.73 years	‐	‐
	SD: 4.96		
	Range: 2 months–25.17 years		

*Note*: Some categories do not add up to 100%, due to small amounts of missing data.

### 3.2 Infant feeding mode

We asked participants to select every type of milk that they had fed to their babies (Table 2). Almost all participants (90.1%) had some experience of breastfeeding and over half (53.9%) had used infant formula, and 116 (76%) participants had some experience of expressing breastmilk.

**Table 2:** Types of milk fed to all babies—Tick all that apply (*n* = 152).

Type of milk	Subtype	*N*	%
Breastmilk (all ways of using your own milk)	‐	137	90.1
Breastmilk	Expressed breastmilk	116	76
Donor human milk	‐	2	1.3
Infant formula (any type)	‐	82	53.9
Infant formula	Powdered infant formula	68	44.7
	Ready‐made liquid infant formula	47	30.9
	Prescription‐only formula	8	5.3
Other	‐	5	3.3

### 3.3 Breastfeeding

The questions presented in Table 3 explored participants' experiences of breastfeeding including motivation, challenges and experience of sensory difficulties during feeds, such as feeling ‘touched out’ (unable to bear being touched further) or experiencing unpleasant sensations. These responses and open text data are reported together below. Of 152 participants, 141 (92.8%) responded to at least one of the two open text questions relating to breastfeeding. In response to the prompt: ‘Please tell us about your experiences of receiving breastfeeding support’, 96 (63.2%) provided information. Furthermore, 71 (46.7%) responded to the question ‘Do you have any recommendations for how breastfeeding support could be made better for Autistic people?’ However, in practice, there was overlap in the contents of each question, with some ‘experiences’ data reported in the ‘recommendations’ question and vice versa.

**Table 3:** Experiences of breastfeeding.

Question	Response categories	*N*	%
How motivated were you to breastfeed your baby, even if you encountered difficulties? (*n* = 133)	Always or most of the time	116	87.2
	About half of the time	7	5.3
	Sometimes	10	7.5
	Never or rarely	0	0
Did you find breastfeeding enjoyable or positive in some way? (*n* = 133)	Always or most of the time	65	48.9
	About half the time	27	20.3
	Sometimes	22	16.5
	Never	18	13.5
	Prefer not to say	1	0.7
How much research did you do to find out about breastfeeding? (*n* = 133)	A great deal or a lot	87	65.4
	A moderate amount	30	22.6
	None or a little	16	12
Did you experience pain when breastfeeding? (*n* = 133)	Always or most of the time	36	27.1
	About half the time	24	18.0
	Sometimes	61	45.9
	Never	11	8.3
	Prefer not to say	1	0.8
Did you express your breastmilk? (*n* = 133)	Always or most of the time	14	10.3
	About half the time	20	15.0
	Sometimes	82	61.7
	Never	17	12.8
Did you find the intensity of breastfeeding difficult? (*n* = 132)	Always or most of the time	62	46.6
	About half the time	22	16.5
	Sometimes	41	30.8
	Never	7	5.3
	Prefer not to say	1	0.8
Did you find the unpredictability of your baby's feeding patterns difficult? (*n* = 133)	Definitely or probably yes	71	53.4
	Might or might not	26	19.5
	Definitely or probably not	36	27.1
Sensory difficulties: Intensity of baby touching my body (*n* = 131)	Always or most of the time	37	28.3
	About half the time	18	13.7
	Sometimes	42	32.1
	Never	34	26.0
Sensory difficulties: Unpleasant sensations from baby suckling (*n* = 132)	Always or most of the time	41	31
	About half the time	20	15.2
	Sometimes	47	35.6
	Never	24	18.2
Sensory difficulties: Unpleasant feeling when milk ‘let down’ (*n* = 133)	Always or most of the time	55	41.4
	About half the time	11	8.3
	Sometimes	40	30.1
	Never	25	18.8

*Note*: Total participants who have experience of breastfeeding *n* = 137, there are fewer in each category due to small amounts of missing data.

#### 3.3.1 Motivation to breastfeed

Motivation to breastfeed, despite any difficulties, was high (87.2%). In the open text responses, 10 participants noted that they were determined to breastfeed. Although this was mostly viewed as positive, two participants identified that they had put a lot of pressure on themselves:

I had very little milk for my first child yet felt so pressured to breastfeed (by myself, not just by others) that I sat for hours pumping to gain a tiny amount—I should have been sleeping (Diagnosed, primarily speaking, white woman with a postgraduate degree aged 52)

#### 3.3.2 Positive breastfeeding experiences

Almost half of the participants (48.9%) noted that they found breastfeeding positive always or most of the time. Despite clear reporting in response to closed questions that breastfeeding was positive for many participants, this was not reflected to the same extent in open text responses, which tended to focus more on challenges. Four participants noted that breastfeeding had become a ‘special interest’ for them; that is, they had become very interested in the topic and that learning about it was a source of intense enjoyment:

I went literally everywhere to get the information I needed and craved about how to feed my babies, I now have an almost encyclopaedic understanding. But so much support was lacking. I had to seek it out. I was dogged though and never gave up. (Diagnosed, primarily AAC user, white woman aged 36 with an undergraduate degree)

In addition, two participants explicitly noted that they did not have *any* negative experiences when breastfeeding:

Breastfeeding was a special interest for a while so I had a lot of information. I was also quite lucky that my baby took to it well and I produced a lot of milk so I didn't need a lot of support from professionals, if any. (Diagnosed, primarily speaking white nonbinary person, aged 32 educated to A' Level)

From a sensory point of view, breastfeeding was also reported by some to be a positive experience:

I found breastfeeding comforting as I could be in my own little bubble without worrying about anything but my son, and I felt the oxytocin from it. (Undergoing diagnosis, primarily speaking, white woman aged 28 with a taught postgraduate degree)

Breastfeeding could also be a source of self‐regulation for some participants who were finding the intensity of new parenthood challenging:

I believe that breastfeeding helped me overcome the feelings of being overwhelmed it stopped me from completely shutting down as I had a purpose and a reason that required me to function at some level. (Undergoing diagnosis, primarily speaking, white woman aged 44 with an NVQ)

However, within the small number of comments focusing on positive sensory experiences, the positives were sometimes caveated by identifying a challenge and how it was overcome:

The sensation of baby's body against mine is wonderful and very calming but I struggle with little hands touching my skin so I wear nursing tops that cover most of my skin now. (Diagnosed, primarily AAC or signing, white woman aged 32 with an undergraduate degree)

In addition to sensory joy, one participant noted that having low sensitivity to touch made managing breastfeeding complications bearable, although they resulted in medical complications:

My hyposensitivity to touch is probably the only reason I managed to breast feed two years. I had latch issues, mastitis, blebs, thrush, biting and pretty much every painful breastfeeding experience. (Diagnosed, primarily writing, white woman aged 38 with a taught postgraduate degree)

#### 3.3.3 Breastfeeding challenges

The research team divided the challenges associated with breastfeeding into two categories. First, those commonly seen in a general population. Second, those that are not typically seen in a general population and/or are known challenges that Autistic people face, for example, due to differences in sensory processing or communication (Beardon, 2019). Breastfeeding issues reported in open text responses that were associated with babies included: baby losing weight (*n* = 11), suspected or diagnosed tongue tie (*n* = 13), cleft palate (*n* = 3) and Gastroesophageal reflux disease (GERD, *n* = 3). Additionally, challenges that frequently relate to receiving inadequate breastfeeding support were reported, including pain (always or most of the time: 27.1%; open text responses: *n* = 18), challenges latching baby on (*n* = 14), bleeding nipples (*n* = 5) and a belief that they had a ‘low supply” of milk (*n* = 5). Less frequently reported challenges included: mastitis, managing cluster feeding and breastfeeding with larger breasts. Four participants reported intense breastfeeding aversion:

I wanted to breastfeed for 2 years and felt awful that I only made it to 14 months. At night I would cry and cry. I just thought I was an awful human being for finding it so hard. But I never shared how hard I found it with anyone at all. For me the sucking was actually ok, but it was the jiggling baby and grabbing at me and (once my children were older) the repetition of limbs flapping up and down against me. (Diagnosed, speaking but prefers written communication, white woman aged 42 with a doctorate)

Autism‐related challenges of breastfeeding were frequently reported and were primarily centred around the bodily differences that Autistic people have, including interoception, proprioception and sensory processing. In response to closed questions, the milk let‐down reflex was challenging for 41.4% of parents, always or most of the time. Five participants described this in more detail:

I had a feeling of dread with every let down so I used a chew necklace and focused on a phone game. (Diagnosed, primarily speaking, white gender fluid person aged 30 with an NVQ)

The sensation of baby suckling was challenging always or most of the time for almost one‐third of participants (31.0%), as one parent explained:

‘I tandem feed my two sons. I find that the intensity with which the older boy suckles is harder to bear’. (Self‐identifying, primarily speaking white woman aged 42 with an undergraduate degree).

Furthermore, 28.3% of parents identified that their baby touching their body was challenging always or most of the time. Five parents described this issue in more detail:

I fed my first till 3.5 and coped well. My second I am feeding at 18 months but I have extreme aversion and my skin crawls sometimes. I have to put her down sometimes as her touching me whilst feeding is too much. (Self‐identifying, primarily speaking white woman aged 28 with an undergraduate degree)

The most commonly reported issue in open text responses (*n* = 9) was that breastfeeding ‘felt odd’, due to interoceptive differences that Autistic people often have. As one parent explained:

It felt like I had an old fashioned telephone ringing in my breasts (don't know how else to describe it). Thinking about it now, I can feel it again and I haven't breastfed in over 9 years. (Diagnosed, primarily speaking, white woman aged 44 with one GCSE obtained as an adult)

The sensation of wet skin from leaking was also identified as being dysregulating in four open text responses. When considering how breastfeeding fit into the Autistic participants' lives, the intensity (46.6%) and unpredictability (53.4%) were identified as challenging always or most of the time for around half of the participants. However, these were not described in detail in the open text responses, with only three parents describing challenges with the intensity:

Breastfeeding was very much a positive experience overall but the intensity was overwhelming at times and the need to be physically present prevented using usual coping strategies of time alone to de‐stress. (Self‐identifying, primarily speaking, white woman aged 37 with an undergraduate degree)

Often when reporting challenges, participants also identified solutions that they had developed to reduce dysregulation. These included modifying clothing so less of the baby's body was touching their body, distraction, such as using a mobile phone to play games or watch videos while breastfeeding, and using nipple shields:

‘Drinking ice water or playing on my phone helped to distract from the unpleasant feelings’. (Diagnosed, primarily speaking white woman aged 37 with an NVQ).

In addition, to prevent dysregulation, some participants noted that they ensured the sensory environment around them was optimised as far as possible and that they did not have conversations with people while feeding.

#### 3.3.4 Expressing

As noted in Table 3, 14 participants (10.3%) stated that they expressed ‘always’ or ‘most of the time’. Despite this, in open text descriptions, most content (*n* = 18) related to expressing was framed negatively, with it described as ‘miserable’, ‘difficult’ and ‘horrendous’. Most comments that identified a particular issue identified a sensory challenge, relating to either the feeling, sound or even both:

‘I hated expressing milk—it made me very angry instantly, I hated the noise and I hated how it felt’. (Diagnosed, primarily speaking white woman aged 40, with a postgraduate degree).

For some, expressing did not produce as much milk as desired, which was frustrating:

My breasts do not respond well to pumps. This was intensely upsetting with my first with my others I was more able to let it go and not put as much emotional energy into building a stash of pumped milk. (Diagnosed, primarily speaking mixed ethnicity woman aged 29 with A' Levels)

However, seven participants reported expressing in more neutral or positive terms, including some who exclusively expressed. Despite expressing enabling some participants to meet their breastfeeding goals, two participants noted that this impacted on their ability to rest and sleep:

I only expressed, it was very structured and I could time everything so I developed a good routine. This was negative though because I was forgetting to eat and not sleeping because I was focusing on expressing and remembering to do that. (Diagnosed, primarily speaking white female aged 25 with an undergraduate degree)

#### 3.3.5 Donor human milk

Two participants noted that they had fed their baby donor human milk. In the open text data, only three participants mentioned donor human milk. One described receiving it, one had donated and a third wished that they had been told about it and been able to receive it while being supported to increase their own breastmilk supply:

I really wanted to breastfeed but couldn't produce enough milk so the baby was starving. I wish they had told me there was such a thing as donated milk but I was just advised to move to formula. I was really sad about that because despite the discomfort I really wanted to do it but I had no support with it. (Diagnosed, primarily speaking white woman aged 44 with A' Levels)

#### 3.3.6 Stopping breastfeeding

Twenty‐seven participants described the circumstances around them stopping breastfeeding. Many decisions to end breastfeeding reportedly began with a baby losing weight or a health professional advising formula top‐ups. Formula top‐ups were viewed negatively by seven participants:

I hated and resented [the move to formula feeding] and it made me feel a failure as well as angry that health care professionals had given me wrong info that led to me feeding her formula. (Diagnosed, primarily writing, white woman aged 36, with an undergraduate degree)

By contrast, six participants noted that they had naturally weaned their child: ‘I was able to breastfeed my son until he self‐weaned at two and a half years old’ (Diagnosed, primarily speaking, nonbinary person aged 42 with a doctorate).

### 3.4 Formula feeding

Next, participants were asked about their experiences of using infant formula, such as selecting brands, preparing feeds and how they felt about their baby's feeding pattern (Table 4). Eighty‐two participants (53.9%) had experience of using formula with their babies, while 70 participants (46.1%) had never fed any of their babies formula. Of the formula feeding participants, 45 (54.9%) responded to the open text question: ‘Is there anything else you would like to tell us about your experiences of feeding your baby infant formula?’ Additionally, 30 participants (36.6%) provided recommendations for how to provide better formula feeding support for Autistic people. Most participants reported that it was easy to select a brand of formula (54.9%) and to understand instructions on how to prepare the formula (68.3%) always or most of the time.

**Table 4:** Experiences of using infant formula.

Question	Response options	*N*	%
Did you find it easy to select a type or brand of infant formula? (*n* = 82)	Yes	45	54.9
	No	20	24.4
	Can't remember	17	20.7
Did you find it easy to understand the instructions on how to make up a bottle of formula? (*n* = 82)	Yes	56	68.3
	No	16	19.5
	Can't remember	9	11.0
	Prefer not to say	1	1.2
Did you find it enjoyable preparing bottles of formula? (*n* = 82)	Extremely or somewhat positive	20	24.4
	Neither positive or negative	32	39.0
	Extremely or somewhat negative	30	36.6
Did you feel anxious about safely preparing formula? (*n* = 82)	Always or most of the time	34	41.5
	About half of the time	9	11.0
	Never or sometimes	37	45.1
	Prefer not to say	2	2.4
Did you find the unpredictability of baby's feeding patterns difficult? (*n* = 82)	Always or most of the time	34	41.5
	About half of the time	4	4.9
	Never or sometimes	40	48.8
	Prefer not to say	4	4.9

Only six participants reported positive aspects relating to formula feeding, which mostly focused on elements of the routine, either preparing bottles or feeding the baby on a schedule. For example:

I found the timed routine of 3–4 hr feeds massively reduced my anxiety over if my baby was receiving enough milk and meant that I could relax and enjoy my time with my babies in the in between times… (and) my partner could do some feeds (which) meant I could have a bath etc. (Diagnosed, primarily speaking white woman aged 41 with A' Levels)

In response to a closed question, over a third of parents (36.6%) noted that preparing the formula was an extremely or somewhat negative experience, and 41.5% felt anxious about preparing the formula safely always or most of the time. Four participants noted that they found the guidance around formula feeding confusing:

Advice is so conflicting and I still don't understand what temperature you should give a baby milk. I was giving it cold from the fridge but started warming it a little now. (Diagnosed, primarily speaking, white female aged 25 with an undergraduate degree)

Participants also identified the executive functioning needed to formula feed as a challenge:

I just found it overwhelming to keep track of making the bottles and keeping everything sterilised and ready. Particularly when I was recovering from a [caesarean] section still. (Diagnosed, primarily speaking white woman aged 52, education level not stated)

This may have been exacerbated by 41.5% of participants finding it difficult to manage the unpredictability of the baby's feeding patterns always or most of the time. To manage these challenges while formula feeding, two parents noted that they did not follow all aspects of safer formula feeding guidance:

We would make up. All. Of the next days bottle in the night and put them in the fridge ready, and reheat…. That was a big help…. A few people. Said it went against current advice at the time but we knew many people with older kids before the current advice changed and they had had no problems with it so we chose to do what worked for us. (Diagnosed, primarily speaking non‐binary person aged 47 with an undergraduate degree)

### 3.5 Infant feeding support

Overall, 75.7% of those with infant feeding experience had received some breastfeeding support. Participants were asked who specifically gave them breastfeeding support (see Table 5). Midwives were the most frequent provider of support (52%), followed by one‐third of people receiving support from health visitors (35.5.%) and the internet (34.2%). Other sources of support reported in the open text responses included reading books about breastfeeding, receiving support from La Leche League and NCT groups and hospital infant feeding specialists.

**Table 5:** Sources of infant feeding support (*n* = 152).

Source	*N*	%
Midwife	79	52.0
Health visitor	54	35.5
Lactation consultant (IBCLC)	37	24.3
Doula	3	2.0
Breastfeeding counsellor	22	14.5
Breastfeeding peer supporter in a one‐to‐one setting	21	13.8
Breastfeeding support group	44	28.9
Friends and family	45	29.6
Internet (such as a Facebook group or forum)	52	34.2
Other	8	5.3

#### 3.5.1 Breastfeeding support

A Kruskal–Wallis test was conducted to identify whether there were any significant differences between parents who had received any type of breastfeeding support or not (yes/no) on their experiences of breastfeeding (using the questions in Table 3). No significant differences were identified between parents who had received any type of breastfeeding support compared to those who had not for any of the breastfeeding experiences questions (*p* > 0.5). Further Kruskal–Wallis tests were then conducted to examine any differences in breastfeeding experiences according to the different types of breastfeeding support received (e.g., peer supporter, lactation consultant, midwife, friends/family, etc). The results showed that receiving certain types of breastfeeding support was associated with significant differences, mainly improvements, in breastfeeding experiences. The significant results are reported below, with the full results of the analysis available in Supporting Information S2: Appendix 1:

1. Overall, the results showed that those who had received support from a health visitor [H(1) = 4.84, *p* = 0.03], lactation consultant [H(1) = 4.62, *p* = 0.03], and one‐to‐one support from a breastfeeding peer supporter [H(1) = 5.40, *p* = 0.02], reported significantly fewer sensory difficulties in relation to the intensity of breastfeeding compared to those who had not received these types of support.
2. Those who had received Health visitor support reported significantly fewer unpleasant sensations from baby sucking [H(1) = 5.56, *p* = 0.02].
3. Those who had attended breastfeeding support groups reported significantly greater motivation to breastfeed [H(1) = 4.38, *p* = 0.04] and greater enjoyment in breastfeeding [H(1) = 8.60, *p* = 0.003].
4. Receiving one‐to‐one support from a peer supporter was associated with doing greater research into breastfeeding [H(1) = 5.79, *p* = 0.02] compared to those who had not had this support.
5. Those who had accessed internet support reported significantly less pain during breastfeeding [H(1) = 6.32, *p* = 0.01] and higher levels of enjoyment in breastfeeding [H(1) = 4.04, *p* = 0.04], compared to those without this support.
6. Participants who had received breastfeeding counsellor support reported higher levels of difficulty with the unpredictability of the baby's feeding patterns [H(1) = 4.23, *p* = 0.04].
7. Support from friends, family and doulas was not found to have significant effects on any of the breastfeeding experience questions.

Overall, these results indicate that breastfeeding support from a range of sources was associated with more positive experiences of breastfeeding. Breastfeeding support was particularly associated positively with difficulties that Autistic parents may be more likely to struggle with such as the intensity of breastfeeding, the unpredictability of baby's feeding patterns and unpleasant sensations from baby sucking.

The majority of open text content relating to infant feeding support came from parents who were breastfeeding. Overall, 23 participants reported positive experiences with at least one health professional supporting breastfeeding, mostly relating to midwives in the community. These participants identified that health professionals were knowledgeable and provided tailored support:

I would not have been able to breast feed without this support. She came to my home regularly to help and give advice. I could call her whenever. She was a great source of information and comfort. (Diagnosed, primarily speaking white woman aged 33 with a taught postgraduate degree)

However, 82 participants reported negative interactions with at least one health professional. These included inadequate support (*n* = 13), providing conflicting information (*n* = 6) and grabbing breasts (*n* = 7). Specific comments noted that services were ‘absent’, ‘lacking’, ‘poor’ ‘rubbish’, ‘outdated’, ‘terrible and unhelpful’. Staff were reported to be: ‘useless’, ‘judgemental’ and to ‘know very little about breastfeeding’. It was also noted that staff ‘didn't listen’ or were unable to understand Autistic communication:

I honestly don't know [what to recommend], but my experience was terrible and things need to change. I'm not sure if they didn't listen to me because of my poor communication or whether they treat everyone like that. (Formally diagnosed, primarily speaking white woman aged 35 with a taught postgraduate degree)

Furthermore, some participants felt their concerns were ‘dismissed’ or that staff failed to understand Autistic sensory needs: ‘Being touched out and [health visitor] and [midwives] not thinking that was a legitimate [reason to struggle with breastfeeding]’. (Self‐identifying, primarily speaking white woman aged 39 with an undergraduate degree). Another participant noted that the breastfeeding support was not tailored to their needs more generally and accordingly was not helpful:

I think I was given a lot of support, however I'm not sure that it really helped as much as it should have. (Self‐identifying, primarily speaking white woman aged 43 with an undergraduate degree)

Specifically focusing on hospital environments, many participants (*n* = 18) noted that they did not receive adequate breastfeeding support. At the worst, participants described painful and upsetting experiences:

Felt like I was dying during the c section and lost a lot of blood but they focused on the baby and not on me and kept commenting how difficult it was coz I was so obese. Took me over 2 hrs to come round and I was too weak to try to feed my baby and had an allergic reaction to the morphine. But they shouted at me for starving my baby and for failing to breast feed. Worst experience of my life. (Diagnosed, primarily speaking, gender non‐conforming, white person aged 45 with an undergraduate degree)

Other participants noted that they did not actually receive in‐hospital breastfeeding support and had to wait for discharge to seek support in the community: ‘Despite being admitted for help with feeding (post‐NICU) I got no help until I left the hospital’ (diagnosed, primarily speaking white woman aged 42 with a doctorate). For some, this arose from finding it extremely difficult to ask for help in a context where help was not proactively offered: ‘Sometimes I felt I couldn't ask for support because other patients were needing attention and pressing their bell’ (diagnosed, primarily speaking white woman aged 34 with an NVQ). The hospital setting was also identified as a barrier to being able to relax and breastfeed, which compounded the issue of inadequate breastfeeding support.

By contrast, occasional references (*n* = 3) were made to receiving excellent support, including from two parents who had a baby in NICU:

Really excellent and hands on support on how to hand express colostrum for a prem baby and how to use a pump by the labour ward midwife. Lots of general support and positioning advice/nipple shield offered by special care staff. Was incredibly useful although I was so determined I would have sought out advice anywhere if it wasn't offered. (Diagnosed, primarily speaking, white woman aged 33 with an NVQ)

Twenty‐one participants described their experiences of breastfeeding support groups, with 15 identifying positive experiences, including tongue ties being identified, signposting to support for cleft palate and being supportive of maternal mental health conditions. This support was identified as ‘reassuring’ and ‘informative’; some participants reported that breastfeeding support groups ‘kept me going’. However, even for those who found breastfeeding support groups valuable for their breastfeeding support, the social interaction could be challenging:

I did not receive any support from health care professionals but attended a few breastfeeding groups. I found the social aspect of the groups difficult so I didn't attend consistently. (Undergoing diagnosis, primarily speaking, white woman aged 30 with an undergraduate degree)

Six participants noted the group interaction style or the communication provided was unhelpful, as it relied on non‐Autistic communication styles and the providing of emotional support only for practical breastfeeding problems. Alternative forms of support included forums and Facebook groups: ‘I have followed a forum and it helped me a lot’. (Diagnosed, primarily speaking Asian woman aged 30 with an undergraduate degree). Furthermore, many participants noted that they did a lot of research themselves to solve their breastfeeding issues.

When asked to provide their recommendations on how to improve breastfeeding support given to Autistic birthing parents, participants had a number of recommendations. Thirty‐eight participants noted that there was a need for those providing breastfeeding support to have a better understanding of Autism, including how it may impact breastfeeding.

Being aware of the sensory issues and having plenty of clear, honest advice. I don't want to have things sugar‐coated. Tell me if it might hurt, be clear and honest. I am less likely to give up if I know what to expect. (Formally diagnosed, primarily speaking white agender person aged 40 with an undergraduate degree)

More specific recommendations focused on using clear and direct communication and making breastfeeding support more accessible, such as not requiring telephone calls or visits to breastfeeding support groups. Providing continuity of carer was also referred to as impacting breastfeeding success by six participants. Alongside this, participants noted general deficiencies of breastfeeding support in the United Kingdom. This included 21 participants stating that those providing breastfeeding support needed better general breastfeeding training, and 13 reporting that there was a need for more breastfeeding support to be made available.

#### 3.5.2 Formula feeding support

Participants were asked whether anyone gave them support with formula feeding their baby: 70% said no, 17.5% said yes, 10% could not remember and 2.5% preferred not to answer. Further Kruskal–Wallis tests were conducted to investigate the potential impact of different types of infant feeding support on participants' experience of using formula. The results showed that those who had received *any* form of breastfeeding support were significantly less likely to use powdered formula compared to participants who had not received any BF support (*χ* ^2^ (1) = 5.33, *p* = 0.02). No other significant differences between parents who had received any form of breastfeeding support or those who had not were identified for any of the formula experience questions.

Kruskal–Wallis tests were then conducted to investigate whether receiving different types of infant feeding support (e.g., midwife support, lactation consultant support) impacted participants' experiences of formula feeding. The full results of these tests can be seen in Supporting Information S1: Appendix 2. The significant associations identified showed that:

1. Those who had received midwife [H(1) = 4.26, *p* = 0.04], internet [H(1) = 6.20, *p* = 0.01] and breastfeeding group support [H(1) = 9.69, *p* = 0.002] were significantly less likely to use powdered infant formula compared to those who had not received these types of support.
2. Participants who had received breastfeeding group support [H(1) = 5.75, *p* = 0.02] and one‐to‐one support from a peer supporter [H(1) = 5.95, *p* = 0.02] were significantly more likely to feel negative about preparing formula bottles compared to those who had not received this support.
3. Those who had received breastfeeding group support [H(1) = 8.91, *p* = 0.003] and internet support [H(1) = 5.02, *p* = 0.03] were significantly more likely to not understand the instructions for preparing formula bottles compared to those who had not received these forms of support.

No significant differences were identified for those who had received health visitor support, lactation consultant support, friend and family support and breastfeeding counsellor support compared to those who had not received any of these types of support for any of the formula experience questions.

Four formula feeding parents noted in the open text boxes that they struggled to get formula feeding advice from their health professionals. Additionally, the primary recommendation regarding formula feeding was to provide more support for formula feeding parents. Eight participants also noted that the format of advice could be improved, such as containing pictures or videos to enhance clarity.

## 4 DISCUSSION

Our survey received responses from 152 Autistic birthing people with first‐hand experience of breastfeeding and/or formula feeding. While the majority (90.1%) of participants had breastfed, only just over half (53.9%) had used infant formula. This is highly unusual in a UK context, where 83% of babies have received infant formula at 3 months and less than one percent of infants are exclusively breastfed to 6 months (Health and Social Care Information Centre, 2012), although selection bias and our avenues of recruitment are likely to have impacted on our data. In the general population, over 80% of birthing parents in the United Kingdom initiate breastfeeding (Health and Social Care Information Centre, 2012), although there is a lack of more specific data on the strength of breastfeeding motivation. Overall, our participants were highly motivated to breastfeed, as has been found in previous research with Autistic parents (Gardner et al., 2016; Hampton et al., 2021). This high level of motivation, alongside known Autistic breastfeeding challenges, could explain the relatively high prevalence (10%) of exclusive, or near exclusive, expressing. Again, we do not have comparable data from a UK general population, but exclusive expressing is typically associated with feeding pre‐term infants and returning to paid employment (Johnson et al., 2013). It has been identified that expressing results in lower milk output and an increased risk of mastitis compared to feeding from the breast (Wong et al., 2017), and that those expressing are less likely to breastfeed exclusively to 6 months (Edmunds et al., 2013). Our participants highlighted sensory challenges relating to expressing that could be considered above those encountered in a general population (Stearns, 2009). However, we did not collect data that would allow a consideration of whether those who expressed met their breastfeeding goals.

Almost half of our participants found breastfeeding positive the majority of the time, which has been found in previous research with Autistic parents (Burton, 2016). That said, several participants noted that they prioritised maintaining breastfeeding at the expense of their own well‐being at times. New parents' belief in their effectiveness as a parent, also known as parenting efficacy, has been associated in a general population with positive outcomes, although lack of parenting efficacy has been associated with post‐natal depression, including feelings of hopelessness, guilt and loss of satisfaction with one's activities (Gross & Marcussen, 2017). Struggling with infant feeding was highlighted by some participants as not only impacting on their parenting efficacy but also increasing feelings of Autistic overwhelm; while others reported being able to meet their breastfeeding goals increased their parenting efficacy. Future trials of breastfeeding support interventions with Autistic parents should consider the impact of interventions on parenting efficacy.

Research in a general population has identified that the desire for a feeding routine can be strong, and symptoms of depression and anxiety can increase if a feeding routine is introduced but is unsuccessful (Harries & Brown, 2019). Furthermore, the unpredictability of feeding routines can lead to parents in a general population stopping breastfeeding (Brown et al., 2011). Both breast and formula feeding parents in our study reported that the intensity and unpredictability of infant feeding routines were challenging to manage. The intensity of new parenthood and loss of previously well‐functioning coping strategies has been previously identified as a challenge for a general population of parents (Doss et al., 2014) as well as Autistic parents (Hampton et al., 2021; Litchman et al., 2019). Sensory issues related to breastfeeding are known to be higher in Autistic people (Hampton et al., 2023), and over 40% of our participants found the milk let‐down reflex unpleasant always or most of the time, highlighting that interoception impacts on breastfeeding experiences. Furthermore, almost one‐third found the baby suckling and/or the intensity of the baby touching their body unpleasant, highlighting the impact of different sensory processing that has been identified in previous research (Quinn, 2021). Despite this, they were still determined to breastfeed, and thus it is important for solutions to be identified where possible to reduce dysregulation, meltdowns or shutdowns.

In a general population of parents in the United Kingdom, support for breastfeeding has often been identified to be inadequate in both quality and availability (Grant et al., 2017). Furthermore, physically Disabled parents from the United Kingdom have reported that health professionals have been unable to give tailored advice (Williams et al., 2019). For parents who are required to take medication, being provided incorrect advice is often reported (Phillips et al., 2018). In the existing Autism research, challenges with accessing breastfeeding support and satisfaction with support provided have been identified (Hampton et al., 2023): our participants did not report any health professional involvement in problem solving for interoceptive and sensory differences. However, our research identified a significant association between receiving breastfeeding support and fewer challenges with the intensity, unpredictability and unpleasant suckling sensations from breastfeeding. By contrast, there was also a significant association between receiving support from a breastfeeding counsellor and increased difficulty with the unpredictability of infant feeding patterns. However, it is not clear if this was the reason for participants accessing support. In addition, our participants reported that Autistic birthing parents could benefit from tailored support, continuity of carer and being able to access support on a one‐to‐one basis. Where postnatal support has previously been provided to Autistic parents, it has not always met their needs and can be an additional source of stress, for example, when appointment times are not honoured and where trust is damaged (Burton, 2016; Dugdale et al., 2021). Support for breast and formula feeding was often reported in open text responses as being inadequate and failing to meet Autistic needs. In particular, there were few reports of good support within the hospital and this is an area in need of urgent improvement. From our data, it was not clear if Autistic parents in hospitals and the community were seeking support from health professionals less than parents in the general population, and this should be considered in future research.

Autistic people have reported breastfeeding support has involved bullying and threats of referral to social work when parents did not want to introduce formula top‐ups because they wished to exclusively breastfeed (Burton, 2016). Social work practice adopts a neuro‐normative lens, which demonises Autistic mothers and uses our neuro difference as a reason for involuntary social work intervention (Benson, 2023). Accordingly, those supporting breastfeeding should troubleshoot breastfeeding issues to support Autistic parents to identify solutions when they are determined to exclusively breastfeed and do not wish to introduce infant formula. It may also be that access to donor human milk would be a more acceptable solution when milk top‐ups are required. Peer‐to‐peer support models or the use of Autistic professionals to design and oversee support provided by non‐Autistic staff may help to ensure Autistic communication and needs are central (Grant, Jones, et al., 2022; Harper, 2019).

In the absence of effective infant feeding support, many participants identified their own strategies to reduce dysregulation when breastfeeding, such as wearing an additional top to reduce the amount of skin‐to‐skin, distracting themselves, such as with their smartphone and sitting in a quiet and sensorily calm room to breastfeed. Research on the general population has identified that smartphone use during breastfeeding does not reduce the quality of mother–child interaction or how responsive the mother is to their infant's needs (Inoue et al., 2022). Accordingly, health professionals should actively encourage Autistic people to use these self‐regulation strategies to support breastfeeding.

Previous research on Autistic parents has not considered formula feeding in detail. Parents in our study reported that it was easy to understand how to prepare infant formula. However, 41.5% of participants reported that preparing the formula was anxiety‐inducing always or most of the time, a higher percentage than has been found in the UK general population (Grant et al., 2023). This may be because of the known executive functioning challenges that many neurodivergent people experience. Infant feeding support for Autistic people should ensure confidence in preparing powdered infant formula and sterilising feeding equipment, including providing supporting materials that can act as a quick reference guide when preparing formula.

### 4.1 Strengths and limitations

This study collected data on the infant feeding experiences of 152 Autistic people from the United Kingdom, adding evidence to the small body of existing research. Our definition of Autistic included those who were diagnosed, undergoing diagnosis and those who self‐identified. This is seen as a strength by Autistic advocates who use a neurodiversity‐affirming paradigm (Walker, 2021), but a weakness by those who adopt a medical model of Autism. Some (*n* = 27, 17.76%) of our participants had given birth over 10 years previously, meaning that there will likely have been changes to the provision of health care since their maternity experience. Furthermore, the mean time since participants' last birth was 6.73 years, and there may therefore have been greater recall bias due to this than in studies with stricter inclusion criteria relating to proximity of birth. In addition, we did not include a comparison group of non‐Autistic participants. Our questions were based on what was already known about Autistic infant feeding experiences from the team's existing systematic review (Grant, Jones, et al., 2022). The sample of participants was likely impacted by self‐selection bias, with a high proportion of highly educated, speaking, cis‐gendered and white participants, limiting the applicability of these findings to Autistic people who are additionally marginalised. In addition, a Facebook group that aims to facilitate Autistic breastfeeding was one recruitment partner, which may have influenced the relatively low levels of formula feeding seen in our data. Furthermore, three participants contacted the lead researcher to say that they wanted to take part but did not have the energy to complete the in‐depth survey, estimated to take up to an hour to complete. These participants shared their experiences by email, with no follow‐up questions to reduce the burden on them, and to ensure that potential participants were not excluded by the data collection methodology. Other eligible people may have decided not to take part because of this issue, and this would have been more likely to occur in Autistic people with high support needs and particularly those who find reading and typing challenging. Furthermore, while we had some community input into our study, it was not in‐depth involvement and did not include those with learning disabilities, which is recommended to ensure that data collection methods in Autism research are accessible and acceptable (Nicolaidis et al., 2019). We did not ask participants about their relationship status, which is known to impact on infant feeding experiences, and should be included in future research on Autistic people.

Our analysis involved multiple comparative tests to explore associations in the data, which may raise the likelihood of a false positive with a significance level of 0.05. That said, our sample size is relatively small meaning that reducing the level of significance to 0.001 may mask potential significant differences in the data. Overall, we recognise the limitations of this but our study is novel and exploratory with the aim of identifying potential differences in experience for Autistic people and highlighting possible avenues for further research and training on this topic area.

### 4.2 Directions for future research

To date, there has been a dearth of research on the infant feeding experiences of Autistic people. This study adds to the small body of research but there is a need for additional in‐depth qualitative research with Autistic people. This should be conducted around or soon after the time that they are breast and formula feeding to reduce recall bias. Existing research has been largely conducted in the United Kingdom (Burton, 2016) and the United States (Litchman et al., 2019), with two studies to date focused on multiple countries (Hampton et al., 2021; Wilson & Andrassy, 2021). Additional research should be undertaken to compare the experiences of Autistic people across countries to shed light on infant feeding support models that may particularly meet Autistic needs. There is also a need for more comparative research between Autistic and non‐Autistic people, as has been conducted by Pohl et al. (2020), and to investigate the infant feeding experiences of a wider group of neurodivergent birthing parents. Future research should be costed appropriately to provide support to those who would like to participate but would find the data collection methods inaccessible (Nicolaidis et al., 2019).

## 5 CONCLUSION

Our research highlights that many Autistic people have a strong desire to breastfeed and almost half found breastfeeding positive always or most of the time. However, Autistic people also frequently reported breastfeeding challenges related to Autistic bodily differences, including sensory processing and interoception. Receiving breastfeeding support was significantly associated with positive breastfeeding experiences, but open text responses highlighted many negative aspects of infant feeding support, and this support did not appear to address Autistic bodily differences that impacted on breastfeeding. There is an urgent need to develop neurodiversity‐affirming breastfeeding support that meets the needs of Autistic people.

## AUTHOR CONTRIBUTIONS

Aimee Grant, Kathryn Williams and Amy Brown were awarded funding for the study, designed the study and developed the data collection tool. Aimee Grant undertook the thematic analysis of open text data. Catrin Griffiths completed the analysis of the quantitative data, with supervision from Amy Brown. Aimee Grant drafted the manuscript. All authors reviewed and agreed on the final manuscript.

## CONFLICT OF INTEREST STATEMENT

Kathryn Williams is the research director for Autistic UK, a Community Interest Company that provides voluntary and paid consultancy. The remaining authors declare no conflict of interest.

## Supporting information

Supporting information.

Supporting information.

## Data Availability

Data are not available for data sharing.

## Footnotes

1 Please note that we capitalise ‘Autistic’ throughout as a marker of culture and identity. Not all Autistic people use this marker, but it can be an important way of claiming our identity, as is explained in Bestow (2021, p. viix). This is similar to the way that some people in the d/Deaf community capitalise ‘Deaf’.
