# Supplementary material for: “It felt like I had an old fashioned telephone ringing in my breasts”: An online survey of UK Autistic birthing parents' experiences of infant feeding
Source: Matern Child Nutr. 2023 Nov 1;20(1):e13581. doi: 10.1111/mcn.13581 (PMC10750003; doi:10.1111/mcn.13581)
Supplement: Supplementary file 1 — Supporting information. [file MCN-20-e13581-s002.docx]

Appendix 2: Infant formula feeding experiences and support received – Kruskall Wallis test results

| *Appendix 2: Table 1: Midwife support and formula experience questions* | | | | |
| --- | --- | --- | --- | --- |
| *Type of feeding support* | *Formula feeding question* | *Test statistic* | *DF* | *Sig* |
| Midwife | Ready made infant formula in bottles | .022 | 1 | .88 |
| Midwife | Powdered that you make with hot water | 4.26 | 1 | .03 * |
| Midwife | Prescription formula | .01 | 1 | .91 |
| Midwife | Unpredictability of baby’s feeding patterns | .04 | 1 | .84 |
| Midwife | Did you feel anxious about safely preparing infant formula? | .06 | 1 | .82 |
| Midwife | How did you find preparing bottles of formula? | .54 | 1 | .46 |
| Midwife | Did you find it easy to understand the instructions on how to make up a bottle of formula? | 2.96 | 1 | .09 |
| Midwife | Did you find it easy to select a type or brand of infant formula? | .48 | 1 | .49 |
| Midwife | Did anybody give you support with learning how to formula feed your baby? | .49 | 1 | .49 |
| Midwife | Did you find breastfeeding enjoyable or positive in some way? | .11 | 1 | .74 |
| *= p>0.05, **<0.01. | | | | |

| *Appendix 2: Table 2: Health Visitor support and formula experience questions* | | | | |  |
| --- | --- | --- | --- | --- | --- |
| *Type of feeding support* | *Formula feeding question* | *Test statistic* | *DF* | *Sig* | |
| Health Visitor | Ready made infant formula in bottles | 1.46 | 1 | .23 | |
| Health Visitor | Powdered that you make with hot water | .16 | 1 | .69 | |
| Health Visitor | Prescription formula | 1.94 | 1 | .16 | |
| Health Visitor | Unpredictability of baby’s feeding patterns | .01 | 1 | .94 | |
| Health Visitor | Did you feel anxious about safely preparing infant formula? | .05 | 1 | .83 | |
| Health Visitor | How did you find preparing bottles of formula? | .00 | 1 | .95 | |
| Health Visitor | Did you find it easy to understand the instructions on how to make up a bottle of formula? | 1.41 | 1 | .24 | |
| Health Visitor | Did you find it easy to select a type or brand of infant formula? | .00 | 1 | .97 | |
| Health Visitor | Did anybody give you support with learning how to formula feed your baby? | .21 | 1 | .65 | |
| Health Visitor | Did you find breastfeeding enjoyable or positive in some way? | 1.91 | 1 | .17 | |
| *= p>0.05, **<0.01. | | | | | |

| *Appendix 2: Table 3: Lactation Consultant support and formula experience questions* | | | | |  |
| --- | --- | --- | --- | --- | --- |
| *Type of feeding support* | *Formula feeding question* | *Test statistic* | *DF* | *Sig* | |
| Lactation consultant | Ready made infant formula in bottles | .40 | 1 | .53 | |
| Lactation consultant | Powdered that you make with hot water | .04 | 1 | .83 | |
| Lactation consultant | Prescription formula | .79 | 1 | .38 | |
| Lactation consultant | Unpredictability of baby’s feeding patterns | .03 | 1 | .85 | |
| Lactation consultant | Did you feel anxious about safely preparing infant formula? | .03 | 1 | .86 | |
| Lactation consultant | How did you find preparing bottles of formula? | 2.48 | 1 | .12 | |
| Lactation consultant | Did you find it easy to understand the instructions on how to make up a bottle of formula? | .32 | 1 | .57 | |
| Lactation consultant | Did you find it easy to select a type or brand of infant formula? | 3.13 | 1 | .08 | |
| Lactation consultant | Did anybody give you support with learning how to formula feed your baby? | .08 | 1 | .78 | |
| Lactation consultant | Did you find breastfeeding enjoyable or positive in some way? | 1.14 | 1 | .29 | |
| *= p>0.05, **<0.01. | | | | | |

| *Appendix 2: Table 4: Doula support and formula experience questions* | | | | |
| --- | --- | --- | --- | --- |
| *Type of feeding support* | *Formula feeding question* | *Test statistic* | *DF* | *Sig* |
| Doula | Ready made infant formula in bottles | 1.82 | 1 | .18 |
| Doula | Powdered that you make with hot water | .16 | 1 | .69 |
| Doula | Prescription formula | .17 | 1 | .68 |
| Doula | Unpredictability of baby’s feeding patterns | 3.70 | 1 | .05 |
| Doula | Did you feel anxious about safely preparing infant formula? | .16 | 1 | .69 |
| Doula | How did you find preparing bottles of formula? | .43 | 1 | .51 |
| Doula | Did you find it easy to understand the instructions on how to make up a bottle of formula? | 2.31 | 1 | .12 |
| Doula | Did you find it easy to select a type or brand of infant formula? | 1.63 | 1 | .20 |
| Doula | Did anybody give you support with learning how to formula feed your baby? | .06 | 1 | .81 |
| Doula | Did you find breastfeeding enjoyable or positive in some way? | .01 | 1 | .04* |
| *= p>0.05, **<0.01. | | | | |

| *Appendix 2: Table 5: BF counsellor support and formula experience questions* | | | | |
| --- | --- | --- | --- | --- |
| *Type of feeding support* | *Formula feeding question* | *Test statistic* | *DF* | *Sig* |
| BF counsellor | Ready made infant formula in bottles | .010 | 1 | .92 |
| BF counsellor | Powdered that you make with hot water | 3.15 | 1 | .07 |
| BF counsellor | Prescription formula | 1.42 | 1 | .23 |
| BF counsellor | Unpredictability of baby’s feeding patterns | .85 | 1 | .36 |
| BF counsellor | Did you feel anxious about safely preparing infant formula? | .20 | 1 | .66 |
| BF counsellor | How did you find preparing bottles of formula? | 1.26 | 1 | .26 |
| BF counsellor | Did you find it easy to understand the instructions on how to make up a bottle of formula? | 1.06 | 1 | .30 |
| BF counsellor | Did you find it easy to select a type or brand of infant formula? | .00 | 1 | .99 |
| BF counsellor | Did anybody give you support with learning how to formula feed your baby? | .92 | 1 | .34 |
| BF counsellor | Did you find breastfeeding enjoyable or positive in some way? | 1.04 | 1 | .31 |
| *= p>0.05, **<0.01. | | | | |

| *Appendix 2: Table 6: BF peer supporter support and formula experience questions* | | | | |
| --- | --- | --- | --- | --- |
| *Type of feeding support* | *Formula feeding question* | *Test statistic* | *DF* | *Sig* |
| BF peer supporter | Ready made infant formula in bottles | .58 | 1 | .45 |
| BF peer supporter | Powdered that you make with hot water | .08 | 1 | .78 |
| BF peer supporter | Prescription formula | .01 | 1 | .91 |
| BF peer supporter | Unpredictability of baby’s feeding patterns | .01 | 1 | .91 |
| BF peer supporter | Did you feel anxious about safely preparing infant formula? | .21 | 1 | .65 |
| BF peer supporter | How did you find preparing bottles of formula? | 5.95 | 1 | .02 * |
| BF peer supporter | Did you find it easy to understand the instructions on how to make up a bottle of formula? | .44 | 1 | .51 |
| BF peer supporter | Did you find it easy to select a type or brand of infant formula? | 3.61 | 1 | .06 |
| BF peer supporter | Did anybody give you support with learning how to formula feed your baby? | .00 | 1 | .97 |
| BF peer supporter | Did you find breastfeeding enjoyable or positive in some way? | .02 | 1 | .89 |
| *= p>0.05, **<0.01. | | | | |

| *Appendix 2: Table 7: BF support group support and formula experience questions* | | | | |
| --- | --- | --- | --- | --- |
| *Type of feeding support* | *Formula feeding question* | *Test statistic* | *DF* | *Sig* |
| BF support group | Ready made infant formula in bottles | .02 | 1 | .88 |
| BF support group | Powdered that you make with hot water | 9.69 | 1 | .00 ** |
| BF support group | Prescription formula | 1.10 | 1 | .29 |
| BF support group | Unpredictability of baby’s feeding patterns | .60 | 1 | .44 |
| BF support group | Did you feel anxious about safely preparing infant formula? | 1.55 | 1 | .21 |
| BF support group | How did you find preparing bottles of formula? | 5.75 | 1 | .02 * |
| BF support group | Did you find it easy to understand the instructions on how to make up a bottle of formula? | 8.91 | 1 | .00 ** |
| BF support group | Did you find it easy to select a type or brand of infant formula? | .67 | 1 | .41 |
| BF support group | Did anybody give you support with learning how to formula feed your baby? | .02 | 1 | .88 |
| BF Support group | Did you find breastfeeding enjoyable or positive in some way? | 8.60 | 1 | .00 ** |
| *= p>0.05, **<0.01. | | | | |

| *Appendix 2: Table 8: Friend and family support and formula experience questions* | | | | |
| --- | --- | --- | --- | --- |
| *Type of feeding support* | *Formula feeding question* | *Test statistic* | *DF* | *Sig* |
| Friends and family support | Ready made infant formula in bottles | .64 | 1 | .42 |
| Friends and family support | Powdered that you make with hot water | 1.24 | 1 | .25 |
| Friends and family support | Prescription formula | 1.18 | 1 | .28 |
| Friends and family support | Unpredictability of baby’s feeding patterns | .00 | 1 | .95 |
| Friends and family support | Did you feel anxious about safely preparing infant formula? | .17 | 1 | .68 |
| Friends and family support | How did you find preparing bottles of formula? | .26 | 1 | .61 |
| Friends and family support | Did you find it easy to understand the instructions on how to make up a bottle of formula? | 3.42 | 1 | .06 |
| Friends and family support | Did you find it easy to select a type or brand of infant formula? | .43 | 1 | .51 |
| Friends and family support | Did anybody give you support with learning how to formula feed your baby? | 3.83 | 1 | .05 |
| Friends and family support | Did you find breastfeeding enjoyable or positive in some way? | 1.00 | 1 | .32 |
| *= p>0.05, **<0.01. | | | | |

| *Appendix 2: Table 9: Internet support and formula experience questions* | | | | |
| --- | --- | --- | --- | --- |
| *Type of feeding support* | *Formula feeding question* | *Test statistic* | *DF* | *Sig* |
| Internet | Ready made infant formula in bottles | .00 | 1 | .98 |
| Internet | Powdered that you make with hot water | 6.20 | 1 | .01* |
| Internet | Prescription formula | .32 | 1 | .57 |
| Internet | Unpredictability of baby’s feeding patterns | .11 | 1 | .74 |
| Internet | Did you feel anxious about safely preparing infant formula? | .02 | 1 | .90 |
| Internet | How did you find preparing bottles of formula? | 3.00 | 1 | .08 |
| Internet | Did you find it easy to understand the instructions on how to make up a bottle of formula? | 5.02 | 1 | .03* |
| Internet | Did you find it easy to select a type or brand of infant formula? | .26 | 1 | .61 |
| Internet | Did anybody give you support with learning how to formula feed your baby? | 1.21 | 1 | .27 |
| Internet | Did you find breastfeeding enjoyable or positive in some way? | 4.04 | 1 | .04* |
| *= p>0.05, **<0.01. | | | | |

| *Appendix 2: Table 10: Other support and formula experience questions* | | | | |
| --- | --- | --- | --- | --- |
| *Type of feeding support* | *Formula feeding question* | *Test statistic* | *DF* | *Sig* |
| Other support | Ready made infant formula in bottles | .14 | 1 | .71 |
| Other support | Powdered that you make with hot water | .18 | 1 | .67 |
| Other support | Prescription formula | .47 | 1 | .50 |
| Other support | Unpredictability of baby’s feeding patterns | 2.13 | 1 | .14 |
| Other support | Did you feel anxious about safely preparing infant formula? | .00 | 1 | .98 |
| Other support | How did you find preparing bottles of formula? | .43 | 1 | .51 |
| Other support | Did you find it easy to understand the instructions on how to make up a bottle of formula? | .01 | 1 | .93 |
| Other support | Did you find it easy to select a type or brand of infant formula? | 2.32 | 1 | .13 |
| Other support | Did anybody give you support with learning how to formula feed your baby? | 4.03 | 1 | .05 |
| Other support | Did you find breastfeeding enjoyable or positive in some way? | .47 | 1 | .49 |
| *= p>0.05, **<0.01. | | | | |

| *Appendix 2: Table 11: Did not receive any formula feeding support and formula experience questions* | | | | |
| --- | --- | --- | --- | --- |
| *Type of feeding support* | *Formula feeding question* | *Test statistic* | *DF* | *Sig* |
| Did not receive any formula feeding support | Ready made infant formula in bottles | .17 | 1 | .68 |
| Did not receive any formula feeding support | Powdered that you make with hot water | 5.14 | 1 | .02* |
| Did not receive any formula feeding support | Prescription formula | .02 | 1 | .90 |
| Did not receive any formula feeding support | Unpredictability of baby’s feeding patterns | .60 | 1 | .44 |
| Did not receive any formula feeding support | Did you feel anxious about safely preparing infant formula? | .25 | 1 | .62 |
| Did not receive any formula feeding support | How did you find preparing bottles of formula? | .34 | 1 | .56 |
| Did not receive any formula feeding support | Did you find it easy to understand the instructions on how to make up a bottle of formula? | 1.24 | 1 | .27 |
| Did not receive any formula feeding support | Did you find it easy to select a type or brand of infant formula? | 4.15 | 1 | .04* |
| Did not receive any formula feeding support | Did anybody give you support with learning how to formula feed your baby? | .26 | 1 | .61 |
| Did not receive any formula feeding support | Did you find breastfeeding enjoyable or positive in some way? | .07 | 1 | .79 |
| *= p>0.05, **<0.01. | | | | |

| *Appendix 2: Table12: Received formula feeding support and formula experience questions* | | | | |
| --- | --- | --- | --- | --- |
| *Type of feeding support* | *Formula feeding question* | *Test statistic* | *DF* | *Sig* |
| Received support with using formula | Ready made infant formula in bottles | 1.29 | 2 | .52 |
| Received support with using formula | Powdered that you make with hot water | 1.30 | 2 | .52 |
| Received support with using formula | Prescription formula | .20 | 2 | .90 |
| Received support with using formula | Unpredictability of baby’s feeding patterns | 1.27 | 2 | .53 |
| Received support with using formula | Did you feel anxious about safely preparing infant formula? | 2.85 | 2 | .24 |
| Received support with using formula | How did you find preparing bottles of formula? | 1.24 | 2 | .54 |
| Received support with using formula | Did you find it easy to understand the instructions on how to make up a bottle of formula? | 1.14 | 2 | .57 |
| Received support with using formula | Did you find it easy to select a type or brand of infant formula? | 1.26 | 2 | .53 |
| Received support with using formula | Did you find breastfeeding enjoyable or positive in some way? | .92 | 2 | .63 |
| *= p>0.05, **<0.01. | | | | |
