# Supplementary material for: “It felt like I had an old fashioned telephone ringing in my breasts”: An online survey of UK Autistic birthing parents' experiences of infant feeding
Source: Matern Child Nutr. 2023 Nov 1;20(1):e13581. doi: 10.1111/mcn.13581 (PMC10750003; doi:10.1111/mcn.13581)
Supplement: Supplementary file 2 — Supporting information. [file MCN-20-e13581-s001.docx]

**Appendix 1: Breastfeeding experiences and support received, Kruskall Wallis test resutls**

| *Appendix 1: Table 1: Midwife support and breastfeeding experience questions* | | | | |
| --- | --- | --- | --- | --- |
| *Type of feeding support* | *Breastfeeding question* | *Test statistic* | *DF* | *Sig* |
| Midwife | Did you find breastfeeding enjoyable or positive in some way? | .11 | 1 | .74 |
| Midwife | How motivated were you to breastfeed your baby (even if you encountered difficulties)? | .11 | 1 | .74 |
| Midwife | How much research did you do to find out about breastfeeding? | 2.38 | 1 | .12 |
| Midwife | Did you experience pain when breastfeeding? | .30 | 1 | .59 |
| Midwife | Did you find the intensity of breastfeeding difficult? | .00 | 1 | .96 |
| Midwife | Did you find the unpredictability of breastfeeding difficult? | .00 | 1 | .97 |
| Midwife | Did you have any sensory difficulties related to the intensity of breastfeeding? | 1.11 | 1 | .29 |
| Midwife | Did you have any unpleasant sensations related to baby sucking when breastfeeding? | .01 | 1 | .92 |
| Midwife | Did you have any unpleasant feeling during milk let down during breastfeeding? | .42 | 1 | .52 |
| Midwife | Did you have any other sensory difficulties during breastfeeding? | .28 | 1 | .60 |
| Midwife | Did you express your breastmilk? | .22 | 1 | .64 |
| *= p>0.05, **<0.01. | | | | |

| *Appendix 1: Table 2: Health visitor support and breastfeeding experience questions* | | | | |
| --- | --- | --- | --- | --- |
| *Type of feeding support* | *Breastfeeding question* | *Test statistic* | *DF* | *Sig* |
| *Health visitor support* | Did you find breastfeeding enjoyable or positive in some way? | 1.91 | 1 | .17 |
| *Health visitor support* | How motivated were you to breastfeed your baby (even if you encountered difficulties)? | 1.53 | 1 | .22 |
| *Health visitor support* | How much research did you do to find out about breastfeeding? | .04 | 1 | .84 |
| *Health visitor support* | Did you experience pain when breastfeeding? | 1.02 | 1 | .31 |
| *Health visitor support* | Did you find the intensity of breastfeeding difficult? | 4.85 | 1 | .03 * |
| *Health visitor support* | Did you find the unpredictability of breastfeeding difficult? | 1.21 | 1 | .27 |
| *Health visitor support* | Did you have any sensory difficulties related to the intensity of breastfeeding? | 4.59 | 1 | .03 * |
| *Health visitor support* | Did you have any unpleasant sensations related to baby sucking when breastfeeding? | 5.56 | 1 | .02 * |
| *Health visitor support* | Did you have any unpleasant feeling during milk let down during breastfeeding? | 2.07 | 1 | .15 |
| *Health visitor support* | Did you have any other sensory difficulties during breastfeeding? | 1.23 | 1 | .27 |
| *Health visitor support* | Did you express your breastmilk? | .25 | 1 | .62 |
| *= p>0.05, **<0.01. | | | | |

| *Appendix 1: Table 3: Lactation consultant support and breastfeeding experience questions* | | | | |
| --- | --- | --- | --- | --- |
| *Type of feeding support* | *Breastfeeding question* | *Test statistic* | *DF* | *Sig* |
| *Lactation consultant support* | Did you find breastfeeding enjoyable or positive in some way? | 1.14 | 1 | .29 |
| *Lactation consultant support* | How motivated were you to breastfeed your baby (even if you encountered difficulties)? | .17 | 1 | .68 |
| *Lactation consultant support* | How much research did you do to find out about breastfeeding? | .42 | 1 | .52 |
| *Lactation consultant support* | Did you experience pain when breastfeeding? | .08 | 1 | .78 |
| *Lactation consultant support* | Did you find the intensity of breastfeeding difficult? | .00 | 1 | .99 |
| *Lactation consultant support* | Did you find the unpredictability of breastfeeding difficult? | .25 | 1 | .62 |
| *Lactation consultant support* | Did you have any sensory difficulties related to the intensity of breastfeeding? | 4.62 | 1 | .03* |
| *Lactation consultant support* | Did you have any unpleasant sensations related to baby sucking when breastfeeding? | .99 | 1 | .32 |
| *Lactation consultant support* | Did you have any unpleasant feeling during milk let down during breastfeeding? | .08 | 1 | .78 |
| *Lactation consultant support* | Did you have any other sensory difficulties during breastfeeding? | .79 | 1 | .38 |
| *Lactation consultant support* | Did you express your breastmilk? | .14 | 1 | .71 |
| *= p>0.05, **<0.01. | | | | |

| *Appendix 1: Table 4: Doula support and breastfeeding experience questions* | | | | |
| --- | --- | --- | --- | --- |
| *Type of feeding support* | *Breastfeeding question* | *Test statistic* | *DF* | *Sig* |
| *Doula support* | Did you find breastfeeding enjoyable or positive in some way? | .01 | 1 | .94 |
| *Doula support* | How motivated were you to breastfeed your baby (even if you encountered difficulties)? | 1.31 | 1 | .25 |
| *Doula support* | How much research did you do to find out about breastfeeding? | .07 | 1 | .79 |
| *Doula support* | Did you experience pain when breastfeeding? | .34 | 1 | .85 |
| *Doula support* | Did you find the intensity of breastfeeding difficult? | 1.93 | 1 | .17 |
| *Doula support* | Did you find the unpredictability of breastfeeding difficult? | 1.28 | 1 | .26 |
| *Doula support* | Did you have any sensory difficulties related to the intensity of breastfeeding? | 1.40 | 1 | .24 |
| *Doula support* | Did you have any unpleasant sensations related to baby sucking when breastfeeding? | .12 | 1 | .73 |
| *Doula support* | Did you have any unpleasant feeling during milk let down during breastfeeding? | .49 | 1 | .49 |
| *Doula support* | Did you have any other sensory difficulties during breastfeeding? | N/A: no one who had received doula support answered |  |  |
| *Doula support* | Did you express your breastmilk? | .20 | 1 | .66 |
| *= p>0.05, **<0.01. | | | | |

| *Appendix 1: Table 5: Breastfeeding counsellor support and breastfeeding experience questions* | | | | |
| --- | --- | --- | --- | --- |
| *Type of feeding support* | *Breastfeeding question* | *Test statistic* | *DF* | *Sig* |
| *Breastfeeding counsellor* | Did you find breastfeeding enjoyable or positive in some way? | 1.04 | 1 | .31 |
| *Breastfeeding counsellor* | How motivated were you to breastfeed your baby (even if you encountered difficulties)? | 1.75 | 1 | .19 |
| *Breastfeeding counsellor* | How much research did you do to find out about breastfeeding? | 4.49 | 1 | .03* |
| *Breastfeeding counsellor* | Did you experience pain when breastfeeding? | .27 | 1 | .61 |
| *Breastfeeding counsellor* | Did you find the intensity of breastfeeding difficult? | .09 | 1 | .77 |
| *Breastfeeding counsellor* | Did you find the unpredictability of breastfeeding difficult? | 4.23 | 1 | .04 * |
| *Breastfeeding counsellor* | Did you have any sensory difficulties related to the intensity of breastfeeding? | .11 | 1 | .74 |
| *Breastfeeding counsellor* | Did you have any unpleasant sensations related to baby sucking when breastfeeding? | .20 | 1 | .89 |
| *Breastfeeding counsellor* | Did you have any unpleasant feeling during milk let down during breastfeeding? | 4.01 | 1 | .05 |
| *Breastfeeding counsellor* | Did you have any other sensory difficulties during breastfeeding? | .04 | 1 | .85 |
| *Breastfeeding counsellor* | Did you express your breastmilk? | .42 | 1 | .52 |
| *= p>0.05, **<0.01. | | | | |

| *Appendix 1: Table 6: Breastfeeding peer supporter and breastfeeding experience questions* | | | | |
| --- | --- | --- | --- | --- |
| *Type of feeding support* | *Breastfeeding question* | *Test statistic* | *DF* | *Sig* |
| *Breastfeeding peer supporter* | Did you find breastfeeding enjoyable or positive in some way? | .02 | 1 | .89 |
| *Breastfeeding peer supporter* | How motivated were you to breastfeed your baby (even if you encountered difficulties)? | 1.30 | 1 | .25 |
| *Breastfeeding peer supporter* | How much research did you do to find out about breastfeeding? | 5.79 | 1 | .02* |
| *Breastfeeding peer supporter* | Did you experience pain when breastfeeding? | .34 | 1 | .56 |
| *Breastfeeding peer supporter* | Did you find the intensity of breastfeeding difficult? | 3.43 | 1 | .06 |
| *Breastfeeding peer supporter* | Did you find the unpredictability of breastfeeding difficult? | 2.08 | 1 | .15 |
| *Breastfeeding peer supporter* | Did you have any sensory difficulties related to the intensity of breastfeeding? | 5.39 | 1 | .02 * |
| *Breastfeeding peer supporter* | Did you have any unpleasant sensations related to baby sucking when breastfeeding? | .69 | 1 | .41 |
| *Breastfeeding peer supporter* | Did you have any unpleasant feeling during milk let down during breastfeeding? | .34 | 1 | .56 |
| *Breastfeeding peer supporter* | Did you have any other sensory difficulties during breastfeeding? | .41 | 1 | .52 |
| *Breastfeeding peer supporter* | Did you express your breastmilk? | .00 | 1 | .95 |
| *= p>0.05, **<0.01. | | | | |

| *Appendix 1: Table 7: Breastfeeding support group and breastfeeding experience questions* | | | | |
| --- | --- | --- | --- | --- |
| *Type of feeding support* | *Breastfeeding question* | *Test statistic* | *DF* | *Sig* |
| *Breastfeeding support group* | Did you find breastfeeding enjoyable or positive in some way? | 8.60 | 1 | .00 ** |
| *Breastfeeding support group* | How motivated were you to breastfeed your baby (even if you encountered difficulties)? | 4.37 | 1 | .04* |
| *Breastfeeding support group* | How much research did you do to find out about breastfeeding? | 2.12 | 1 | .15 |
| *Breastfeeding support group* | Did you experience pain when breastfeeding? | .54 | 1 | .46 |
| *Breastfeeding support group* | Did you find the intensity of breastfeeding difficult? | 2.71 | 1 | .10 |
| *Breastfeeding support group* | Did you find the unpredictability of breastfeeding difficult? | .25 | 1 | .62 |
| *Breastfeeding support group* | Did you have any sensory difficulties related to the intensity of breastfeeding? | .02 | 1 | .90 |
| *Breastfeeding support group* | Did you have any unpleasant sensations related to baby sucking when breastfeeding? | .78 | 1 | .38 |
| *Breastfeeding support group* | Did you have any unpleasant feeling during milk let down during breastfeeding? | 4.00 | 1 | .05 |
| *Breastfeeding support group* | Did you have any other sensory difficulties during breastfeeding? | .47 | 1 | .49 |
| *Breastfeeding support group* | Did you express your breastmilk? | .04 | 1 | .85 |
| *= p>0.05, **<0.01. | | | | |

| *Appendix 1: Table 8: Friends and family support and breastfeeding experience questions* | | | | |
| --- | --- | --- | --- | --- |
| *Type of feeding support* | *Breastfeeding question* | *Test statistic* | *DF* | *Sig* |
| *Friends and family support* | Did you find breastfeeding enjoyable or positive in some way? | 1.00 | 1 | .32 |
| *Friends and family support* | How motivated were you to breastfeed your baby (even if you encountered difficulties)? | 2.07 | 1 | .15 |
| *Friends and family support* | How much research did you do to find out about breastfeeding? | 2.60 | 1 | .11 |
| *Friends and family support* | Did you experience pain when breastfeeding? | .78 | 1 | .38 |
| *Friends and family support* | Did you find the intensity of breastfeeding difficult? | .92 | 1 | .34 |
| *Friends and family support* | Did you find the unpredictability of breastfeeding difficult? | 1.52 | 1 | .22 |
| *Friends and family support* | Did you have any sensory difficulties related to the intensity of breastfeeding? | 1.23 | 1 | .26 |
| *Friends and family support* | Did you have any unpleasant sensations related to baby sucking when breastfeeding? | .00 | 1 | .95 |
| *Friends and family support* | Did you have any unpleasant feeling during milk let down during breastfeeding? | .40 | 1 | .53 |
| *Friends and family support* | Did you have any other sensory difficulties during breastfeeding? | .62 | 1 | .43 |
| *Friends and family support* | Did you express your breastmilk? | .03 | 1 | .85 |
| *= p>0.05, **<0.01. | | | | |

| *Appendix 1: Table 9: Internet support and breastfeeding experience questions* | | | | |
| --- | --- | --- | --- | --- |
| *Type of feeding support* | *Breastfeeding question* | *Test statistic* | *DF* | *Sig* |
| *Internet support* | Did you find breastfeeding enjoyable or positive in some way? | 4.04 | 1 | .04* |
| *Internet support* | How motivated were you to breastfeed your baby (even if you encountered difficulties)? | 3.72 | 1 | .05 |
| *Internet support* | How much research did you do to find out about breastfeeding? | 2.36 | 1 | .13 |
| *Internet support* | Did you experience pain when breastfeeding? | 6.32 | 1 | .01 * |
| *Internet support* | Did you find the intensity of breastfeeding difficult? | .49 | 1 | .48 |
| *Internet support* | Did you find the unpredictability of breastfeeding difficult? | .80 | 1 | .37 |
| *Internet support* | Did you have any sensory difficulties related to the intensity of breastfeeding? | .27 | 1 | .60 |
| *Internet support* | Did you have any unpleasant sensations related to baby sucking when breastfeeding? | .01 | 1 | .93 |
| *Internet support* | Did you have any unpleasant feeling during milk let down during breastfeeding? | .00 | 1 | .98 |
| *Internet support* | Did you have any other sensory difficulties during breastfeeding? | 1.13 | 1 | .29 |
| *Internet support* | Did you express your breastmilk? | 1.74 | 1 | .19 |
| *= p>0.05, **<0.01. | | | | |

| *Appendix 1: Table 10: Other support and breastfeeding experience questions* | | | | |
| --- | --- | --- | --- | --- |
| *Type of feeding support* | *Breastfeeding question* | *Test statistic* | *DF* | *Sig* |
| *Other support* | Did you find breastfeeding enjoyable or positive in some way? | .47 | 1 | .49 |
| *Other support* | How motivated were you to breastfeed your baby (even if you encountered difficulties)? | .91 | 1 | .34 |
| *Other support* | How much research did you do to find out about breastfeeding? | 1.46 | 1 | .23 |
| *Other support* | Did you experience pain when breastfeeding? | .23 | 1 | .64 |
| *Other support* | Did you find the intensity of breastfeeding difficult? | .17 | 1 | .68 |
| *Other support* | Did you find the unpredictability of breastfeeding difficult? | .30 | 1 | .59 |
| *Other support* | Did you have any sensory difficulties related to the intensity of breastfeeding? | 1.28 | 1 | .26 |
| *Other support* | Did you have any unpleasant sensations related to baby sucking when breastfeeding? | .18 | 1 | .68 |
| *Other support* | Did you have any unpleasant feeling during milk let down during breastfeeding? | 2.36 | 1 | .13 |
| *Other support* | Did you have any other sensory difficulties during breastfeeding? | .01 | 1 | .91 |
| *Other support* | Did you express your breastmilk? | .50 | 1 | .48 |
| *= p>0.05, **<0.01. | | | | |

| *Appendix 1: Table 11: No breastfeeding support and breastfeeding experience questions* | | | | |
| --- | --- | --- | --- | --- |
| *Type of feeding support* | *Breastfeeding question* | *Test statistic* | *DF* | *Sig* |
| *No breastfeeding support* | Did you find breastfeeding enjoyable or positive in some way? | .07 | 1 | .79 |
| *No breastfeeding support* | How motivated were you to breastfeed your baby (even if you encountered difficulties)? | .27 | 1 | .60 |
| *No breastfeeding support* | How much research did you do to find out about breastfeeding? | .18 | 1 | .67 |
| *No breastfeeding support* | Did you experience pain when breastfeeding? | .70 | 1 | .40 |
| *No breastfeeding support* | Did you find the intensity of breastfeeding difficult? | .24 | 1 | .63 |
| *No breastfeeding support* | Did you find the unpredictability of breastfeeding difficult? | .01 | 1 | .93 |
| *No breastfeeding support* | Did you have any sensory difficulties related to the intensity of breastfeeding? | 1.59 | 1 | .21 |
| *No breastfeeding support* | Did you have any unpleasant sensations related to baby sucking when breastfeeding? | .19 | 1 | .66 |
| *No breastfeeding support* | Did you have any unpleasant feeling during milk let down during breastfeeding? | 1.69 | 1 | .19 |
| *No breastfeeding support* | Did you have any other sensory difficulties during breastfeeding? | 2.20 | 1 | .14 |
| *No breastfeeding support* | Did you express your breastmilk? | 2.91 | 1 | .09 |
| *= p>0.05, **<0.01. | | | | |

| *Appendix 1: Table 12:* Any type of breastfeeding support *and breastfeeding experience questions* | | | | |
| --- | --- | --- | --- | --- |
| *Type of feeding support* | *Breastfeeding question* | *Test statistic* | *DF* | *Sig* |
| Any type of breastfeeding support | Did you find breastfeeding enjoyable or positive in some way? | 1.21 | 1 | .27 |
| Any type of breastfeeding support | How motivated were you to breastfeed your baby (even if you encountered difficulties)? | .32 | 1 | .57 |
| Any type of breastfeeding support | How much research did you do to find out about breastfeeding? | .67 | 1 | .41 |
| Any type of breastfeeding support | Did you experience pain when breastfeeding? | .86 | 1 | .35 |
| Any type of breastfeeding support | Did you find the intensity of breastfeeding difficult? | .06 | 1 | .81 |
| Any type of breastfeeding support | Did you find the unpredictability of breastfeeding difficult? | .01 | 1 | .91 |
| Any type of breastfeeding support | Did you have any sensory difficulties related to the intensity of breastfeeding? | 2.02 | 1 | .16 |
| Any type of breastfeeding support | Did you have any unpleasant sensations related to baby sucking when breastfeeding? | .01 | 1 | .93 |
| Any type of breastfeeding support | Did you have any unpleasant feeling during milk let down during breastfeeding? | .28 | 1 | .60 |
| Any type of breastfeeding support | Did you have any other sensory difficulties during breastfeeding? | 2.20 | 1 | .14 |
| Any type of breastfeeding support | Did you express your breastmilk? | 2.48 | 1 | .12 |
| *= p>0.05, **<0.01. | | | | |
